# Assessment of surveillance predictors for suspected respiratory syncytial virus, influenza and *Streptococcus pneumoniae* infections in children aged <5 years in Madagascar

**DOI:** 10.1016/j.ijregi.2021.12.003

**Published:** 2021-12-13

**Authors:** Norosoa Harline Razanajatovo, Zo Zafitsara Andrianirina, Todisoa Andriatahina, Julia Guillebaud, Aina Harimanana, Elisoa Hariniaina Ratsima, Hervé Rakotoariniaina, Arnaud Orelle, Rila Ratovoson, Judickaelle Irinantenaina, Dina Arinalina Rakotonanahary, Lovasoa Ramparany, Frédérique Randrianirina, Jean-Michel Heraud, Vincent Richard

**Affiliations:** a National Influenza Centre, Virology Unit, Institut Pasteur de Madagascar, Antananarivo, Madagascar; b Paediatric Ward, Centre Hospitalier Soavinandriana, Antananarivo, Madagascar; c Paediatric Ward, Centre Hospitalier de District de Moramanga, Antananarivo, Madagascar; d Epidemiology and Clinical Research Unit, Institut Pasteur de Madagascar, Antananarivo, Madagascar; e Clinical and Medical Laboratory, Institut Pasteur de Madagascar, Antananarivo, Madagascar; f International Division, Institut Pasteur International Network, Institut Pasteur, Paris, France

**Keywords:** Acute respiratory infections, Syndromic approach, Children, Madagascar

## Abstract

**Background:**

The lack of rapid, sensitive and affordable diagnostic tests that can distinguish a wide variety of respiratory pathogens at the point of care is an obstacle to the rapid implementation of control measures following events and epidemics. In addition, the absence of a standardized case definition to differentiate putative aetiologies is a challenge to assessing the burden of disease. This study aimed to identify the clinical spectrum of respiratory pathogens commonly associated with respiratory tract infections in the context of disease surveillance.

**Methods:**

Data obtained from prospective hospital-based severe acute respiratory infection surveillance among children aged <5 years from November 2010 to July 2013 were used in this study.

**Results:**

Intercostal recession and dyspnoea were predictive of respiratory **syncytial** virus (RSV) infection, whereas headache and chills were more often observed during influenza A infection. Male patients were at a higher risk for RSV infection than female patients. Productive cough, chills, sweating and weight loss were significantly associated with *Streptococcus pneumoniae* infection. The presence of fever did not necessarily indicate RSV infection.

**Conclusions:**

Combined with other examinations, this study shows the value of including the syndromic approach in the panel of diagnostic criteria for rapid identification of the risk of infectious diseases in areas where laboratory diagnostics are challenging. Given the current situation with coronavirus disease 2019, this approach may help decision makers to implement appropriate control measures.

## Highlights

• A sensitive surveillance case definition can be defined based on specific symptoms.
• Intercostal recession and dyspnoea may be used to identify children with respiratory syncytial virus (RSV) infection.
• Fever was not necessarily a good predictor of paediatric RSV infection.
• Headache may be used to identify influenza infection in children.
• Sweats and productive cough may define infection due to *Streptococcus pneumoniae* in children.

## Introduction

Lower respiratory tract infections (LRTIs) are a leading cause of morbidity and mortality, particularly in children aged <5 years. Indeed, the Global Burden of Disease Study conducted in 2015 showed that LRTIs caused more than 2.7 million deaths worldwide, making them the fifth leading cause of deaths overall and the leading cause of deaths in children aged <5 years (GBD 2015 LRI Collaborators, 2017). Most of these deaths occurred in low- and middle-income countries (LIMCs), where access to health care and treatment is limited (Izadnegahdar et al., 2013).

In LIMCs, approximately 11 million children aged <5 years were admitted for severe acute respiratory infections (SARIs) in 2010, with an estimated case fatality ratio of 2.3%; this compares with 570,000 cases and a case fatality ratio of 0.6% in developed countries (Nair et al., 2011). Although respiratory syncytial viruses (RSV) are known to be the leading cause of viral pneumonia in children (Benet et al., 2017), there are still knowledge gaps regarding the epidemiology of RSV in developing countries.

A number of studies have confirmed the high incidence of respiratory viruses in children hospitalized for a SARI in Africa (Feikin et al., 2012, 2013; Breiman et al., 2015; McMorrow et al., 2015; Rha et al., 2019). Published studies have reported that >70% of cases of respiratory infection in children aged <5 years in Madagascar were associated with viral infection (Hoffmann et al., 2012; Razanajatovo et al., 2018), with an estimated incidence of RSV-associated hospitalization of 11,299 per year (Rabarison et al., 2019).

One of the major challenges in assessing the burden of LRTIs is the lack of a standardized case definition in systems mainly focused on influenza surveillance (Breiman et al., 2013). Symptoms of RSV disease can vary and differ from those of influenza (Hall et al., 2013). In a previous study by the present authors, RSV infections likely caused rhinorrhoea among outpatients presenting with influenza-like illness compared with influenza infections (Razanajatovo et al., 2011). The aim of this study was to identify the clinical predictors of the main respiratory pathogens frequently detected among hospitalized children aged <5 years during SARI surveillance to standardize surveillance case definitions, and contribute to the monitoring of adapted control measures in the absence of laboratory data.

## Materials and methods

Information concerning the study design has been published previously (Razanajatovo et al., 2018).

### Study sites

Prospective hospital-based SARI surveillance was conducted from November 2010 to July 2013 at two selected sites in Madagascar: the University Hospital of Soavinandriana (CENHOSOA), located in the capital city of Antananarivo, and the District Hospital (CHD II) in Moramanga. CENHOSOA is a national referral hospital and is among the four largest hospitals serving the 2.5 million inhabitants of Antananarivo. CHD II is the only local referral hospital for the health district of Moramanga, serving approximately 250,000 inhabitants. It is located 115 km east of Antananarivo. Moramanga encompasses both semi-urban and rural areas.

### Study subjects

For children aged <5 years, the eligibility criteria were suspected sepsis or SARI diagnosed by a physician. The World Health Organization (WHO)-modified case definition for SARI was used, which included bronchiolitis, pneumonia, bronchitis, pleural effusion, cough and difficulty breathing, as published previously (Rajatonirina et al., 2013). Onset of illness had to have been <7 days prior to hospitalization. For each consenting patient, demographic, socio-economic, clinical and epidemiological data were recorded on case report forms (CRFs).

### Biological analysis

Nasopharyngeal, blood and sputum specimens were collected for each enrolled patient and shipped to the Institut Pasteur de Madagascar laboratories, where they were processed immediately or stored at 4°C until testing (tests were performed within 48 h post-sampling). All procedures for biological analyses have been described previously (Rajatonirina et al., 2013; Razanajatovo et al., 2018). Briefly, nasopharyngeal swabs were screened for 14 respiratory viruses using a previously reported in-house multiplex real-time polymerase chain reaction assay (Razanajatovo et al., 2018). Sputum was collected for cytobacteriological testing and blood samples were collected for blood cell counts. For young children, sputum was obtained by nasopharyngeal aspiration.

### Data analysis

Mono-infection was defined as an infection caused by one pathogen (virus or bacteria), and multiple infection was defined as an infection caused by two or more pathogens in a single specimen. Univariate analysis and logistic regression were performed using R software. In univariate analysis, qualitative variables were compared using Fisher's exact test or Chi-squared test. Logistic regressions were performed to adjust odds ratios (OR_a_) found via maximum-likelihood estimation according to infection status for each dependent variable. Variables were compared using the Wald test. *P*≤0.05 was considered to indicate significance.

### Ethics statement

This study was approved by the National Ethics Committee of the Ministry of Health in Madagascar (Authorization N °068-MSANP/CE). For all children, consent was obtained from the parents or legal guardians. Parents were fully informed of the study objectives and procedures, and written informed consent was obtained before enrolment in the study. After obtaining the consent/assent form, the survey team proceeded to collect samples and complete the CRFs. Refusal to consent and prior hospitalization during the 2 weeks preceding the consultation were reasons for exclusion.

## Results

### Description of the population and clinical signs

From November 2010 to July 2013, 693 children aged <5 years presenting to the two study hospitals with SARI were included in this study. Among them, 33.8% (234/693) were aged <12 months and 74.1% (513/693) were aged <24 months. The sex ratio (male/female) was 1.2. No significant differences in age distribution were found between genders (*P*=0.72, Chi-squared test) (Table 1).

**Table 1:** Demographic and clinical characteristics of children aged <5 years hospitalized for sepsis or severe acute respiratory infection in Madagascar from November 2010 to July 2013.

Gender			Total		Female		Male	
		*n*	(%)	*n*	(%)	*n*	(%)	*P*-value
		693	(100.0)	315	(45.5)	378	(54.5)	
Age group (years)								
0–1		234	(33.8)	99	(31.4)	135	(35.7)	0.72
1–2		279	(40.3)	131	(41.6)	148	(39.2)	
2–3		101	(14.6)	46	(14.6)	55	(14.6)	
3–4		54	(7.8)	28	(8.9)	26	(6.9)	
4–5		25	(3.6)	11	(3.5)	14	(3.7)	
Symptoms								
Fever	Yes	411	(60.7)	190	(61.7)	221	(59.9)	0.63
(*n*=677)	No	266	(39.3)	118	(38.3)	148	(40.1)	
Dry cough	Yes	314	(46.5)	141	(45.8)	173	(47.1)	0.75
(*n*=675)	No	361	(53.5)	167	(54.2)	194	(52.9)	
Productive cough	Yes	353	(52.3)	164	(53.1)	189	(51.6)	0.75
(*n*=675)	No	322	(47.7)	145	(46.9)	177	(48.4)	
Dyspnoea	Yes	570	(84.1)	258	(83.5)	312	(84.6)	0.75
(*n*=678)	No	108	(15.9)	51	(16.5)	57	(15.4)	
Chest pain	Yes	35	(6.1)	10	(3.9)	25	(8.0)	0.05
(n =571)	No	536	(93.9)	249	(96.1)	287	(92.0)	
Runny nose	Yes	517	(76.5)	238	(77.5)	279	(75.6)	0.58
(*n*=676)	No	159	(23.5)	69	(24.4)	95	(22.5)	
Sore throat	Yes	54	(9.0)	26	(9.5)	28	(8.5)	0.67
(*n*=601)	No	547	(91.0)	247	(90.5)	300	(91.5)	
Headache	Yes	26	(4.5)	14	(5.4)	12	(3.8)	0.42
(*n*=573)	No	547	(95.5)	245	(94.6)	302	(96.2)	
Chills	Yes	57	(8.5)	24	(7.9)	33	(9.0)	0.67
(*n*=669)	No	612	(91.5)	280	(92.1)	332	(91.0)	
Sweating	Yes	152	(22.6)	71	(23.3)	81	(22.0)	0.71
(*n*=673)	No	521	(77.4)	234	(76.7)	287	(78.0)	
Anorexia	Yes	314	(46.4)	145	(47.4)	169	(45.7)	0.69
(*n*=676)	No	362	(53.6)	161	(52.6)	201	(54.3)	
Vomiting	Yes	158	(23.3)	70	(22.7)	88	(23.8)	0.78
(*n*=678)	No	520	(76.7)	238	(77.3)	282	(76.2)	
Diarrhoea	Yes	86	(12.7)	41	(13.4)	45	(12.2)	0.73
(*n*=676)	No	590	(87.3)	266	(86.2)	324	(87.8)	
Weight loss	Yes	161	(24.0)	77	(25.5)	84	(22.8)	0.47
(*n*=670)	No	509	(76.0)	225	(74.5)	284	(77.2)	
Asthenia	Yes	289	(43.1)	136	(44.9)	153	(41.6)	0.39
(*n*=671)	No	382	(56.9)	167	(55.1)	215	(58.4)	
GPD	Yes	166	(24.6)	80	(26.0)	86	(23.4)	0.47
(*n*=676)	No	510	(75.4)	228	(74.0)	282	(76.6)	
Intercostal recession	Yes	507	(75.2)	226	(73.9)	281	(76.4)	0.47
(*n*=674)	No	167	(24.8)	80	(26.1)	87	(23.6)	
MNW	Yes	360	(53.6)	154	(50.5)	206	(56.1)	0.16
(*n*=672)	No	312	(46.4)	151	(49.5)	161	(43.9)	
Cyanosis	Yes	99	(14.8)	39	(12.8)	60	(16.4)	0.23
(*n*=670)	No	571	(85.2)	265	(87.2)	306	(83.6)	

GPD, general physical deterioration; MNW, movement of nose wings; *n*, number of patients that responded with ‘yes’ or ‘no’ for a given symptom.

The most common clinical signs found among patients with SARIs were dyspnoea (84.1%, 570/678), runny nose (76.5%, 517/676), intercostal recession (75.2%, 507/674), fever (60.7%, 411/677), productive cough (52.3%, 353/675) and dry cough (46.5, 314/675) (Table 1). No significant differences in clinical signs were found between genders.

### Aetiology

Among the tested children, 84.3% (584/693) were positive for at least one pathogen: most (55.3%, 323/584) were laboratory-confirmed positive for RSV, followed by *S. pneumoniae* (26.4%, 154/584) and influenza virus (25.9%, 151/584) (Table S1, see online supplementary material). Viruses were detected more frequently than bacterial pathogens.

Children aged <24 months were infected by RSV significantly more often than children aged >24 months [49.9% (256/513) vs 38.9% (70/180); OR 1.52, 95% confidence interval (CI) 1.07–2.19; *P*=0.02] (Table 2). No significant differences in influenza A (*P*=0.10) or *S. pneumoniae* infections (*P*=0.15) (data not shown) were found between age groups. Male patients were at higher risk for RSV infection than female patients [50.8 (192/378) vs 41.6% (131/315); *P*=0.02] (Table 2).

**Table 2:** Comparison of respiratory syncytial virus (RSV) infection versus non-RSV infection in Madagascar from November 2010 to July 2013.

		RSV infection		Non-RSV infection		Risk factors					
	*n*	*n*	(%)	*n*	(%)	OR	95% CI	*P*-value	OR_a_	95% CI	*P*-value
Sites											
Cenhosoa	488	244	(50.0)	244	(50.0)	1.00	ref				
Moramanga	205	79	(38.5)	126	(61.5)	0.62	0.44–0.88	<0.01	*		
Gender											
Male	378	192	(50.8)	186	(49.2)	1.00	ref				
Female	315	131	(41.6)	184	(58.4)	0.69	0.50–0.94	0.02	*		
Age (years)											
0–2	513	253	(49.3)	260	(50.7)	1.52	1.07–2.19	0.02	*		
2–5	180	70	(38.9)	110	(61.1)	1.00	ref				
Symptoms											
Fever	677	181	(56.9)	230	(64.1)	0.74	0.54–1.01	0.06	*		
Dry cough	675	148	(46.8)	166	(46.2)	1.02	0.76–1.39	0.88			
Productive cough	675	165	(52.4)	188	(52.2)	1.01	0.74–1.36	0.97			
Dyspnoea	678	281	(88.4)	289	(80.3)	1.87	1.22–2.89	<0.01	*		
Chest pain	571	10	(3.8)	25	(8.1)	0.45	0.20–0.93	0.04	*		
Runny nose	676	243	(76.7)	274	(76.3)	1.02	0.71–1.46	0.92			
Sore throat	601	22	(8.0)	32	(9.8)	0.79	0.44–1.39	0.42			
Headache	573	4	(1.5)	22	(7.1)	0.20	0.06–0.53	<0.01	*		
Chills	669	10	(3.2)	47	(13.2)	0.22	0.10–0.42	<0.01	0.36	0.16–0.72	<0.01
Sweating	673	42	(13.4)	110	(30.6)	0.35	0.24–0.52	<0.01	0.43	0.28–0.65	<0.01
Anorexia	676	153	(48.3)	161	(44.8)	1.15	0.85–1.55	0.37			
Vomiting	678	80	(25.2)	78	(21.7)	1.22	0.85–1.74	0.28			
Diarrhoea	676	46	(14.5)	40	(11.2)	1.34	0.85–2.12	0.20			
Weight loss	670	57	(18.4)	104	(28.9)	0.55	0.38–0.80	<0.01	*		
Asthenia	671	120	(38.6)	169	(46.9)	0.71	0.52–0.97	<0.03	*		
GPD	676	68	(21.5)	98	(27.2)	0.73	0.51–1.04	0.08	*		
Intercostal recession	674	264	(83.3)	243	(68.1)	2.34	1.62–3.40	<0.01	2.15	1.47–3.16	<0.01
MNW	672	174	(55.4)	186	(52.0)	1.15	0.85–1.56	0.37			
Cyanosis	670	55	(17.5)	44	(12.4)	1.51	0.98–2.32	0.06	*		

GPD, general physical deterioration; MNW, movement of nose wings; OR, crude odds ratio, OR_a_, adjusted odds ratio; CI, confidence interval; ref, reference.

⁎ Variables included in the initial model.

Mono-infection occurred in 42.3% (293/693) of cases, of which 53.9% (158/293) were RSV (49% of total RSV infections), 7.8% (23/293) were influenza A (20% of total influenza A infections), and 6% (18/293) were *S. pneumoniae* (12% of total *S. pneumoniae* infections). Comparison of RSV infection (mono-infection group and multiple infection group) with non-RSV infection showed that the risk for RSV infection was higher in children aged <24 months for mono-infections (OR_a_ 1.48, 95% CI 0.94–2.37) and multiple infections (OR_a_ 2.42, 95% CI 1.42–4.26) (Tables 3 and 4). No significant differences were found between genders. An analysis of influenza virus A and *S. pneumoniae* showed no differences between age groups or between genders (Tables S2 and S3, see online supplementary material).

**Table 3:** Comparison of respiratory syncytial virus (RSV) mono-infection vs non-RSV infection in Madagascar from November 2010 to July 2013.

		RSV mono-infection		Non-RSV infection		Risk factors					
	*n*	*n*	(%)	*n*	(%)	OR	95% CI	*P*-value	OR_a_	95% CI	*P*-value
Sites											
Cenhosoa	361	117	(32.4)	244	(67.6)	1.00	ref				
Moramanga	167	41	(24.6)	126	(75.4)	0.68	0.44–1.05	0.08	*		
Gender											
Male	279	93	(33.3)	186	(66.7)	1.00	ref				
Female	249	65	(26.1)	184	(73.9)	0.70	0.47–1.05	0.07	*		
Age (years)											
0–2	383	123	(48.9)	260	(51.1)	1.48	0.94–2.37	0.08	*		
2–5	145	35	(37.6)	110	(62.4)	1.00	ref				
Symptoms											
Fever	513	80	(51.9)	230	(64.1)	0.61	0.41–0.89	0.01	*		
Dry cough	512	68	(44.4)	166	(46.2)	0.93	0.63–1.36	0.71			
Productive cough	512	84	(55.3)	188	(52.2)	1.13	0.77–1.66	0.53			
Dyspnoea	514	134	(87.0)	289	(80.3)	1.65	0.98–2.88	0.07	*		
Chest pain	424	4	(3.5)	25	(8.1)	0.41	0.12–1.08	0.10	*		
Runny nose	512	113	(73.9)	274	(76.3)	0.88	0.57–1.36	0.55			
Sore throat	450	7	(5.6)	32	(9.8)	0.54	0.22–1.20	0.16	*		
Headache	426	3	(2.5)	22	(7.1)	0.34	0.08–1.00	0.08	*		
Chills	506	2	(1.3)	47	(13.2)	0.09	0.01–0.30	<0.01	0.15	0.02–0.52	0.01
Sweating	510	19	(12.7)	110	(30.6)	0.33	0.19–0.55	<0.01	0.43	0.24–0.74	<0.01
Anorexia	512	71	(46.4)	161	(44.8)	1.06	0.73–1.56	0.75			
Vomiting	514	37	(24.0)	78	(21.7)	1.14	0.73–1.78	0.56			
Diarrhoea	512	19	(12.3)	40	(11.2)	1.12	0.61–1.98	0.70			
Weight loss	510	31	(20.7)	104	(28.9)	0.64	0.40–1.00	0.06	*		
Asthenia	510	58	(38.7)	169	(46.9)	0.71	0.48–1.05	0.09	*		
GPD	514	31	(20.1)	98	(27.2)	0.67	0.42–1.05	0.09	*		
Intercostal recession	510	129	(84.3)	243	(68.1)	2.52	1.57–4.19	<0.01	2.18	1.34–3.67	<0 .01
MNW	508	84	(56.0)	186	(52.0)	1.18	0.80–1.73	0.41			
Cyanosis	506	23	(15.3)	44	(12.4)	1.28	0.73–2.20	0.37			

GPD, general physical deterioration; MNW, movement of nose wings; OR, crude odds ratio; OR_a_, adjusted odds ratio; CI, confidence interval; ref, reference.

⁎ Variables included in the initial model.

**Table 4:** Comparison of respiratory syncytial virus (RSV) infection versus non-RSV infection among the 293 cases of multiple infections in Madagascar from November 2010 to July 2013.

		RSV infection		Non-RSV infection				
	*n*	*n*	(%)	*n*	(%)	OR	95% CI	*P*-value
Sites								
Cenhosoa	225	127	(56.4)	98	(43.6)	1.00	ref	
Moramanga	68	38	(55.9)	30	(44.1)	0.98	0.55–1.76	0.99
Gender								
Male	166	99	(59.6)	67	(40.3)	1.00	ref	
Female	127	66	(51.9)	61	(48.1)	0.73	0.44–1.19	0.19
Age (years)								
0–2	207	130	(62.8)	77	(37.2)	**2.42**	**1.42–4.26**	**<0.01**
2–5	86	35	(40.7)	51	(59.3)	1.00	ref	
Symptoms								
Fever	291	101	(61.6)	88	(69.3)	0.71	0.43–1.16	0.17
Dry cough	291	80	(49.1)	49	(38.3)	1.55	0.97–2.50	0.07
Productive cough	291	81	(49.7)	78	(60.9)	0.63	0.39–1.01	0.06
Dyspnoea	192	147	(89.6)	110	(85.9)	1.41	0.70–2.89	0.34
Chest pain	264	6	(4.1)	10	(8.5)	0.46	0.15–1.27	0.13
Runny nose	291	130	(79.3)	104	(81.9)	0.85	0.46–1.52	0.57
Sore throat	273	15	(9.9)	18	(14.8)	0.64	0.30–1.32	0.23
Headache	265	1	(0.7)	6	(5.1)	**0.13**	**0.01–0.76**	**0.05**
Chills	288	8	(4.9)	23	(18.4)	**0.23**	**0.09–0.51**	**<0.01**
Sweating	291	23	(14.1)	46	(35.9)	**0.29**	**0.16–0.51**	**<0.01**
Anorexia	292	82	(50.0)	66	(51.6)	0.94	0.59–1.49	0.79
Vomiting	292	43	(26.2)	35	(27.3)	0.94	0.56–1.60	0.83
Diarrhoea	291	27	(16.5)	17	(13.4)	1.28	0.67–2.50	0.47
Weight loss	288	26	(16.2)	41	(32.0)	**0.41**	**0.23–0.72**	**<0.01**
Asthenia	289	63	(38.5)	70	(54.7)	**0.52**	**0.32–0.83**	**<0.01**
GPD	290	37	(22.8)	40	(31.2)	0.65	0.38–1.10	0.11
Intercostal recession	291	135	(82.3)	88	(69.3)	**2.06**	**1.19–3.60**	**0.01**
MNW	291	90	(54.9)	68	(53.5)	1.06	0.66–1.68	0.86
Cyanosis	291	32	(19.5)	14	(11.0)	**1.96**	**1.01–3.95**	**0.05**

GPD, general physical deterioration; MNW, movement of nose wings; OR, crude odds ratio; CI, confidence interval; ref, reference.

### Clinical signs associated with RSV infection

Compared with influenza A, children with RSV infection were at higher risk for intercostal recession, with OR_a_ of 2.06 (95% CI 1.19–3.60) for multiple infections (Table 4) and OR_a_ of 5.37 (95% CI 2.29–13.90) for mono-infections (Table 5). In addition, dyspnoea (OR_a_ 1.87, 95% CI 1.22–2.89) was detected more often in patients with RSV infection, whereas chest pain (OR_a_ 0.45, 95% CI 0.20–0.93), headache (OR_a_ 0.20, 95% CI 0.06–0.53), chills (OR_a_ 0.22, 95% CI 0.10–0.42), sweating (OR_a_ 0.35, 95% CI 0.24–0.52), weight loss (OR_a_ 0.55, 95% CI 0.38–0.80) and asthenia (OR_a_ 0.71, 95% CI 0.52–0.97) were detected less often in patients with RSV infection (Table 2). Cyanosis was significantly associated with multiple infections involving RSV (*P*=0.05) (Table 4). On the other hand, headache was significantly associated with influenza A infection (*P*=0.03**)** (Table 5). Sweating (*P*<0.01) and productive cough (*P*=0.02) were found more often in patients with *S. pneumoniae* infection (Tables S3 and S4, see online supplementary material). Chills (*P*<0.01) could indicate both influenza A and *S. pneumoniae* infections (Tables 5 and 6).

**Table 5:** Comparison of respiratory syncytial virus (RSV) mono-infection and influenza A mono-infection in Madagascar from November 2010 to July 2013.

		RSV		Influenza A				
	*n*	*n*	(%)	*n*	(%)	OR	95% CI	*P*-value
Sites								
Cenhosoa	131	117	(89.3)	14	(10.7)	1.00	ref	
Moramanga	50	41	(82.0)	9	(18.0)	0.55	0.20–1.54	0.21
Gender								
Male	101	93	(92.1)	8	(7.9)	1.00	ref	
Female	80	65	(81.2)	15	(18.8)	0.37	0.13–1.00	0.05
Age (years)								
0–1	59	51	(86.4)	8	(13.6)	1.00	Ref	0.46
1–2	81	72	(88.9)	9	(11.1)	1.25	0.44–3.50	
2–3	24	21	(87.5)	3	(12.5)	1.10	0.29–5.38	
3–4	10	7	(70.0)	3	(30.0)	0.37	0.08–1.96	
4–5	7	7	(100)	0	(0.0)	–	—	
Symptoms								
Fever	176	80	(51.9)	16	(72.7)	0.41	0.14–1.04	0.07
Dry cough	175	68	(44.4)	9	(40.9)	1.16	0.47–2.95	0.75
Productive cough	175	84	(55.3)	15	(65.2)	0.66	0.25–1.61	0.37
Dyspnoea	177	134	(87.0)	17	(73.9)	2.36	0.78–6.47	0.11
Chest pain	136	4	(3.5)	2	(9.5)	0.34	0.06–2.59	0.24
Runny nose	176	113	(73.9)	18	(78.3)	0.78	0.25–2.12	0.65
Sore throat	146	7	(5.6)	2	(9.5)	0.56	0.12–3.97	0.49
Headache	139	3	(2.5)	3	(14.3)	**0.16**	**0.03–0.90**	**0.03**
Chills	172	2	(1.3)	4	(17.4)	**0.06**	**0.01–0.35**	**<0.01**
Sweating	173	19	(12.7)	4	(17.4)	0.69	0.23–2.56	0.54
Anorexia	176	71	(46.4)	7	(30.4)	1.98	0.80–5.40	0.17
Vomiting	177	37	(24.0)	2	(8.7)	3.32	0.91–21.4	0.12
Diarrhoea	177	19	(12.3)	0	(0.0)	–		
Weight loss	173	31	(20.7)	4	(17.4)	1.24	0.43–4.50	0.72
Asthenia	173	58	(38.7)	10	(43.5)	0.82	0.34–2.04	0.66
GPD	177	31	(20.1)	5	(21.7)	0.91	0.33–2.92	0.85
Intercostal recession	175	129	(84.3)	11	(50.0)	**5.37**	**2.29–13.9**	**<0.01**
MNW	172	84	(56.0)	9	(40.9)	1.84	0.75–4.70	0.19
Cyanosis	171	23	(15.3)	2	(9.5)	1.72	0.46–11.3	0.49

GPD, general physical deterioration; MNW, movement of nose wings; OR, crude odds ratio; CI, confidence interval; ref, reference.

**Table 6:** Comparison of respiratory syncytial virus (RSV) mono-infection and *Streptococcus pneumoniae* mono-infection in Madagascar from November 2010 to July 2013.

		RSV		*S. pneumoniae*				
	*n*	*n*	(%)	*n*	(%)	OR	95% CI	*P*-value
Sites								
Cenhosoa	129	117	(90.7)	12	(9.3)	1.00	ref	
Moramanga	47	41	(87.2)	6	(12.8)	0.70	0.22–2.43	0.57
Gender								
Male	105	93	(88.6)	12	(11.4)	1.00	ref	
Female	71	65	(91.5)	6	(8.5)	1.39	0.46–4.77	0.61
Age (years)								
0–1	57	51	(89.5)	6	(10.5)	1.00	ref	0.25
1–2	79	72	(91.1)	7	(8.9)	1.21	0.37–3.85	
2–3	22	21	(95.5)	1	(4.5)	2.47	0.39–48.2	
3–4	10	7	(70.0)	3	(30.0)	0.27	0.06–1.52	
4–5	8	7	(87.5)	1	(12.5)	0.82	0.11–16.8	
Symptoms								
Fever	172	80	(51.9)	9	(50.0)	1.08	0.40–2.91	0.88
Dry cough	171	67	(44.4)	6	(33.3)	1.60	0.59–4.80	0.37
Productive cough	170	84	(55.3)	14	(77.8)	0.35	0.10–1.04	0.08
Dyspnoea	172	134	(87.0)	15	(83.3)	1.34	0.29–4.52	0.66
Chest pain	132	4	(3.5)	2	(11.8)	0.27	0.05–2.07	0.15
Runny nose	171	113	(73.9)	14	(77.8)	0.81	0.22–2.40	0.72
Sore throat	142	7	(5.6)	1	(5.9)	0.95	0.15–18.4	0.96
Headache	134	3	(2.5)	1	(6.2)	0.39	0.05–8.19	0.43
Chills	167	2	(1.3)	3	(16.7)	**0.07**	**0.01–0.44**	**<0.01**
Sweating	167	19	(12.7)	7	(41.2)	**0.21**	**0.07–0.63**	**<0.01**
Anorexia	170	71	(46.4)	10	(58.8)	0.61	0.21–1.66	0.33
Vomiting	171	37	(24.0)	2	(11.8)	2.37	0.63–15.5	0.27
Diarrhoea	171	19	(12.3)	2	(11.8)	1.06	0.27–7.03	0.95
Weight loss	167	31	(20.7)	8	(47.1)	**0.29**	**0.11–0.84**	**0.02**
Asthenia	167	58	(38.7)	8	(47.1)	0.71	0.26–1.99	0.50
GPD	171	31	(20.1)	7	(41.2)	0.36	0.13–1.06	0.06
Intercostal recession	171	129	(84.3)	10	(55.6)	**4.30**	**1.50–12.5**	**<0.01**
MNW	168	84	(56.0)	10	(55.6)	1.02	0.37–2.72	0.97
Cyanosis	168	23	(15.3)	2	(11.1)	1.45	0.38–9.55	0.64

GPD, general physical deterioration; MNW, movement of nose wings; OR, crude odds ratio; CI, confidence interval; ref, reference.

## Discussion

This study examined the clinical characteristics of pathogens frequently detected during SARIs, and highlighted the need to understand the clinical spectrum associated with each pathogen to establish a more sensitive surveillance case definition for rapid identification of the causative agents and improve surveillance.

It is obvious that basic therapeutic orientation and case management by clinicians cannot be based on clinical indicators of viral infections alone. However, such indicators used in a specific epidemiological context (e.g. active circulation of RSV) could assist clinicians in their decision making, such as starting or delaying antibiotic therapy. In the absence of point-of-care testing and with limited access to a diagnostic laboratory in low-income countries, the use of a specific definition of SARI could aid rapid implementation of strict isolation measures to avoid nosocomial infections within paediatric departments. Nonetheless, this study also supports the need to implement rapid diagnostic tests for rapid adoption of therapeutic measures.

Among recorded respiratory symptoms, intercostal recession and dyspnoea were found more often in patients with RSV infections, and headache was more common in patients with influenza A infection. Sweating and productive cough may be predictive of *S. pneumoniae* infection*.* These results show that the clinical presentation of each pathogen may differ depending on the acute respiratory infection.

Many studies have shown the role of viral pathogens in childhood pneumonia, including multiple infections that also involve bacteria (Ruuskanen et al., 1999; Juven et al., 2000; McIntosh, 2002; Choi et al., 2006; Nichols et al., 2008; Watt et al., 2009; Feikin et al., 2012). The differentiation of pathogens that cause pneumonia among children aged <5 years is challenging without laboratory diagnostics and is often expensive. It is clear that multiplex assays can rapidly identify the presence of many key organisms simultaneously from respiratory specimens (Krause et al., 2014). Although they have great promise for improving the diagnosis of pneumonia, they are still poorly accessible in LIMCs, where the incidence of SARIs is highest, supporting the neeed to establish syndromic surveillance. Indeed, strengthening clinical skills and knowledge of semiology is still useful in settings without technological platforms for diagnosis. The syndromic approach is important not only for orienting diagnostics but also to define respiratory infection surveillance criteria that target influenza viruses and viruses of pandemic risk, which are among the major worldwide threats.

Better characterization of the RSV disease burden in a variety of settings is a priority, as several vaccines, immunoprophylaxis therapies and antiviral drugs to prevent and treat RSV are currently in development (Mazur et al., 2015). A major challenge in RSV surveillance is the lack of a uniform case definition for identifying the disease. Indeed, it is often difficult to distinguish RSV from other respiratory viruses based on its clinical presentation (Razanajatovo et al., 2011, 2018). In the present study, the clinical spectrum of RSV infection differed from that of influenza and *S. pneumoniae.* Indeed, intercostal recession and dyspnoea were significantly associated with RSV infection. In addition, children aged <2 years were at higher risk for RSV infection. In agreement with the results of (Emma et al., 2019), fever was not necessarily found to be predictive of confirmed RSV infection. It would be relevant to remove fever to identify all RSV cases in children aged <5 years (Rha et al., 2019). Taking this into consideration, the case definitions used in the authors’ previous study that included fever (Razanajatovo et al., 2018) would be less sensitive for the detection of RSV infection, leading to potential underestimation of the RSV disease burden.

Although RSV represents a substantial proportion of the SARI burden (Berkley et al., 2010; Breiman et al., 2015; Simpson et al., 2016), respiratory symptoms are characteristically non-specific, even among hospitalized children, for whom the spectrum of possible causative agents is large. It would be of great interest to consider unusual clinical signs during severe illness to deal with this challenge, especially if intercostal recession and, eventually, dyspnoea are present.

This study had several limitations. First, clinical information was not collected correctly for all children. As it was difficult to identify certain clinical symptoms, such as sore throat and headache in younger children, the observed prevalence may have been overestimated, leading to bias in the statistical analysis. In addition, all clinical signs were not available or were uncertain for a number of cases. The data were not considered in these cases. Furthermore, the inclusion criteria were based on WHO's case definition for SARI, and inclusion in the study depended on the physician's diagnosis and admitting practices, for which clinical judgement may have differed. Misclassification bias occurred due to a number of included cases with incomplete or non-documented clinical information. The clinical spectrum for each pathogen may vary between age groups. Thus, there is a need to analyse clinical symptoms according to age. To avoid any bias, other non-clinical variables, such as radiography and oxygen therapy, were not included in this study as they are not common practice at the hospital sentinel sites, are not used for epidemiological surveillance, and are mainly used only for children whose parents can pay. The study data were collected almost 10 years ago, and the prevalence rates of respiratory viruses, including RSV and influenza, may have changed since then. However, it was considered that clinical presentation and case definition have remained the same for common respiratory viruses. Moreover, respiratory infections continue to be a public health problem, and all data contribute to the development of better strategies to reduce the burden of disease caused by acute respiratory illnesses.

In conclusion, a number of clinical signs can be used as surveillance predictors for specific infections among children aged <5 years. Combined with other clinical diagnostic approaches, the method described here could be informative for the rapid implementation of control measures in the absence of laboratory data. As coronavirus disease 2019 is integrated into SARI surveillance, the syndromic approach may help as a proxy for rapid differentiation of causative agents and control the risk of infection through rapid patient management. Research to address challenges in the aetiological diagnosis of SARIs in low-resource countries, and widespread implementation of treatment interventions beyond vaccines and antibiotics are necessary to improve surveillance and early warning systems, and mitigate the burden of SARIs and the impact on child survival within the context of the Sustainable Development Goals.

## Declaration of Competing Interest

None of the authors have financial or personal conflicts of interest related to this study. The corresponding author has full access to all data in the study and takes final responsibility for the decision to submit this publication.

## Appendix Supplementary materials

Image, application 1
