# Supplementary material for: Assessment of surveillance predictors for suspected respiratory syncytial virus, influenza and Streptococcus pneumoniae infections in children aged <5 years in Madagascar
Source: IJID Reg. 2021 Dec 13;2:82–9. doi: 10.1016/j.ijregi.2021.12.003 (PMC9216384; doi:10.1016/j.ijregi.2021.12.003)
Supplement: Supplementary file 1 [file mmc1.docx]

**S1 Table. Etiological agents and multiple infections among children below 5 years of age hospitalized for SARI in Madagascar from November 2010 to July 2013**

|  | **FLUA** | **FLUB** | **COV-OC43** | **COV-NL63** | **COV-229E** | **COV-HKU1** | **RSV** | **HMPV** | **RV** | **PIV1** | **PIV2** | **PIV3** | **ADV** | **BOV** | ***S. pneumoniae*** | **Hib** | **Strepto** | | |
| --- | --- | --- | --- | --- | --- | --- | --- | --- | --- | --- | --- | --- | --- | --- | --- | --- | --- | --- | --- |
| **FLUA** | **23** | 14 | 2 | 0 | 1 | 0 | 47 | 6 | 14 | 0 | 0 | 1 | 8 | 6 | 29 | 9 | 7 |  |  |
| **FLUB** | - | **10** | 0 | 2 | 0 | 0 | 12 | 1 | 4 | 0 | 0 | 0 | 4 | 3 | 11 | 6 | 2 |  |  |
| **COV-OC43** | - | - | **2** | 1 | 0 | 0 | 7 | 1 | 2 | 0 | 3 | 0 | 2 | 2 | 6 | 0 | 1 |  |  |
| **COV-NL63** | - | - | - | **3** | 0 | 0 | 5 | 0 | 3 | 0 | 0 | 0 | 1 | 1 | 4 | 2 | 0 |  |  |
| **COV-229E** | - | - | - | - | **1** | 0 | 1 | 0 | 0 | 0 | 0 | 0 | 0 | 0 | 0 | 0 | 0 |  |  |
| **COV-HKU1** | - | - | - | - | - | 1 | 0 | 0 | 0 | 0 | 0 | 0 | 0 | 0 | 0 | 0 | 0 |  |  |
| **RSV** | - | - | - | - | - | - | **158** | 3 | 35 | 1 | 1 | 0 | 24 | 14 | 66 | 31 | 6 |  |  |
| **HMPV** | - | - | - | - | - | - | - | **7** | 1 | 1 | 1 | 0 | 0 | 0 | 11 | 5 | 1 |  |  |
| **RV** | - | - | - | - | - | - | - | - | **28** | 1 | 3 | 0 | 15 | 11 | 31 | 10 | 2 |  |  |
| **PIV1** | - | - | - | - | - | - | - | - | - | **1** | 0 | 0 | 2 | 1 | 2 | 2 | 2 |  |  |
| **PIV2** | - | - | - | - | - | - | - | - | - | - | **4** | 0 | 0 | 0 | 2 | 2 | 1 |  |  |
| **PIV3** | - | - | - | - | - | - | - | - | - | - | - | **2** | 0 | 1 | 2 | 1 | 0 |  |  |
| **ADV** | - | - | - | - | - | - | - | - | - | - | - | - | **15** | 7 | 20 | 6 | 3 |  |  |
| **BOV** | - | - | - | - | - | - | - | - | - | - | - | - | - | **5** | 12 | 3 | 0 |  |  |
| ***S. pneumoniae*** | - | - | - | - | - | - | - | - | - | - | - | - | - | - | **18** | 29 | 0 |  |  |
| **Hib** | - | - | - | - | - | - | - | - | - | - | - | - | - | - | - | **9** | 3 |  |  |
| **Strepto** | - | - | - | - | - | - | - | - | - | - | - | - | - | - | - | - | **4** |  |  |
| **Monoinf** | 23 | 10 | 2 | 3 | 1 | 1 | 158 | 7 | 28 | 1 | 4 | 2 | 15 | 5 | 18 | 9 | 5 |  |  |
| **2 pathogens** | 51 | 7 | 10 | 5 | 2 | 0 | 101 | 13 | 37 | 3 | 2 | 5 | 28 | 7 | 73 | 21 | 11 |  |  |
| **3 pathogens** | 30 | 12 | 5 | 2 | 0 | 0 | 44 | 9 | 24 | 3 | 4 | 0 | 15 | 13 | 43 | 26 | 7 |  |  |
| **4 pathogens** | 8 | 5 | 1 | 2 | 0 | 0 | 16 | 0 | 13 | 1 | 1 | 0 | 7 | 5 | 15 | 9 | 1 |  |  |
| **5 pathogens** | 1 | 2 | 1 | 1 | 0 | 0 | 4 | 0 | 2 | 0 | 0 | 0 | 2 | 2 | 4 | 1 | 0 |  |  |
| **6 pathogens** | 1 | 1 | 0 | 0 | 0 | 0 | 0 | 0 | 0 | 0 | 0 | 0 | 1 | 1 | 1 | 1 | 0 |  |  |
| **Total** | **114** | **37** | **19** | **13** | **3** | **1** | **323** | **29** | **104** | **8** | **11** | **7** | **68** | **33** | **154** | **67** | **23** |  |  |

FLUA: influenza virus A; FLUB: influenza virus B; COV: coronavirus; RSV: respiratory syncytial virus; HMPV: human metapneumovirus; RV: rhinovirus; PIV: parainfluenza virus; ADV: adenovirus; BOV: bocavirus; *S*. *pneumoniae*: *Streptocccus pneumoniae*; Hib: *Haemophilus influenzae* type b; Strepto: other species of *Streptococcus*.

**S2 Table. Clinical signs relative to influenza A mono-infection in Madagascar from November 2010 to July 2013**

|  |  | **Influenza A** | | **No Influenza A** | | **Risk factors** | | | | | | | | | | | |  |  |
| --- | --- | --- | --- | --- | --- | --- | --- | --- | --- | --- | --- | --- | --- | --- | --- | --- | --- | --- | --- |
|  | **N** | n | (%) | n | (%) | OR^†^ | | 95%CI | | | p-value | | aOR^‡^ | | 95%CI | | p-value | | |
| **Sites** |  |  |  |  |  |  | |  | | |  | |  | |  | |  | | |
| Cenhosoa | 417 | 14 | (3.4) | 403 | (96.7) | 1.00 | | ref | |  | |  | |  | |  | | |  |
| Moramanga | 185 | 9 | (4.9) | 176 | (95.1) | 1.47 | | [0.55 - 3.73] | | 0.36 | |  | |  | |  | | |  |
| **Gender** |  |  |  | | | | |  | |  | |  | |  | |  | | |  |
| Male | 327 | 8 | (2.4) | 319 | (97.6) | 1.00 | | ref | |  | |  | |  | |  | | |  |
| Female | 275 | 15 | (5.4) | 260 | (94.6) | 2.29 | | [0.89 - 6.36] | | 0.08 | | 1.89 | | [0.75 - 5.02] | | 0.18 | | |  |
| **Age** |  |  |  |  |  |  | |  | |  | |  | |  | |  | | |  |
| [0 - 1 yr] | 201 | 8 | (4.0) | 193 | (96.0) | | 1.00 | | ref | 0.77 | |  | |  | |  | | |  |
| [1 - 2 yr] | 253 | 9 | (3.6) | 244 | (96.4) | | 0.89 | | [0.33 - 2.41] |  | |  | |  | |  | | |  |
| [2 - 3 yr] | 84 | 3 | (3.6) | 81 | (96.4) | | 0.89 | | [0.19 - 3.18] |  | |  | |  | |  | | |  |
| [3 - 4 yr] | 43 | 3 | (7.0) | 40 | (93.0) | | 1.81 | | [0.38 - 6.57] |  | |  | |  | |  | | |  |
| [4 - 5 yr] | 21 | 0 | (0.0) | 21 | (100.0) | | 0.00 | | -- |  | |  | |  | |  | | |  |
| **Symptoms** |  |  |  |  |  |  | |  | |  | |  | |  | |  | | |  |
| Fever | 587 | 16 | (72.7) | 336 | (59.5) | 1.82 | | [0.74 - 5.13] | | 0.22 | |  | |  | |  | | |  |
| Dry cough | 585 | 9 | (40.9) | 265 | (47.1) | 0.78 | | [0.32 - 1.83] | | 0.57 | |  | |  | |  | | |  |
| Productive cough | 585 | 15 | (65.2) | 289 | (51.4) | 1.77 | | [0.76 - 4.46] | | 0.20 | | 1.26 | | [0.50 - 3.34] | | 0.63 | | |  |
| Dyspnea | 588 | 17 | (73.9) | 475 | (84.1) | 0.54 | | [0.22 - 1.52] | | 0.20 | | 0.51 | | [0.19 - 1.56] | | 0.21 | | |  |
| Chest pain | 489 | 2 | (9.5) | 25 | (5.3) | 1.87 | | [0.29 - 6.93] | | 0.42 | |  | |  | |  | | |  |
| Runny nose | 587 | 18 | (78.3) | 425 | (75.4) | 1.18 | | [0.46 - 3.62] | | 0.75 | |  | |  | |  | | |  |
| Sore throat | 516 | 2 | (9.5) | 44 | (8.9) | 1.08 | | [0.17 - 3.88] | | 0.92 | |  | |  | |  | | |  |
| Headache | 491 | 3 | (14.3) | 20 | (4.3) | 3.75 | | [0.83 - 12.3] | | 0.05 | | 2.03 | | [0.29 - 8.69] | | 0.39 | | |  |
| Chills | 583 | 4 | (17.4) | 42 | (7.5) | 2.60 | | [0.73 - 7.29] | | 0.10 | | 1.16 | | [0.31 - 3.57] | | 0.81 | | |  |
| Sweating | 584 | 4 | (17.4) | 119 | (21.2) | 0.78 | | [0.22 - 2.13] | | 0.66 | |  | |  | |  | | |  |
| Anorexia | 586 | 7 | (30.4) | 253 | (44.9) | 0.54 | | [0.20 - 1.28] | | 0.18 | | 0.48 | | [0.15 - 1.30] | | 0.17 | | |  |
| Vomiting | 588 | 2 | (8.7) | 128 | (22.7) | 0.33 | | [0.05 - 1.13] | | 0.13 | | 0.36 | | [0.05 - 1.34] | | 0.19 | | |  |
| Diarrhea | 586 | 0 | -- | 76 | (13.5) | 0 | | --- | | 0.98 | |  | |  | |  | | |  |
| Weight loss | 581 | 4 | (17.4) | 126 | (22.6) | 0.72 | | [0.21 - 1.96] | | 0.56 | |  | |  | |  | | |  |
| Asthenia | 582 | 10 | (43.5) | 230 | (41.1) | 1.10 | | [0.46 - 2.54] | | 0.82 | |  | |  | |  | | |  |
| GPD | 587 | 5 | (21.7) | 133 | (23.6) | 0.90 | | [0.29 - 2.31] | | 0.84 | |  | |  | |  | | |  |
| Intercostal recession | 585 | 11 | (50.0) | 427 | (75.8) | **0.32** | | **[0.13 - 0.76]** | | **0.01** | | **0.32** | | **[0.12 - 0.82]** | | **0.02** | | |  |
| MNW | 583 | 9 | (40.9) | 301 | (53.7) | 0.60 | | [0.24 - 1.41] | | 0.24 | |  | |  | |  | | |  |
| Cyanosis | 581 | 2 | (9.5) | 81 | (14.5) | 0.62 | | [0.10 - 2.20] | | 0.53 | |  | |  | |  | | |  |

GPD: general physical deterioration; MNW: movement of nose wings

^†^OR=crude odd ratio; ^‡^aOR= adjusted odd ratio; ***variables included in the initial model

**S3 Table. Clinical signs relative to *S. pneumoniae* mono-infection in Madagascar from November 2010 to July 2013**

|  |  | **Mono-infection** | | **No *S. Pneumoniae*** | | **Risk Factors** | | | | | |
| --- | --- | --- | --- | --- | --- | --- | --- | --- | --- | --- | --- |
|  | **N** | n | (%) | n | (%) | OR^†^ | 95%CI | p-value | aOR^‡^ | 95%CI | p-value |
| **Sites** |  |  |  |  |  |  |  |  |  |  |  |
| Cenhosoa | 375 | 12 | (3.2) | 363 | (96.8) | 1.00 | ref |  |  |  |  |
| Moramanga | 182 | 6 | (3.3) | 176 | (96.7) | 1.03 | [0.31 - 3.03] | 0.99 |  |  |  |
| **Gender** |  |  |  |  |  |  |  |  |  |  |  |
| Male | 303 | 12 | (3.9) | 291 | (96.1) | 1.00 | ref |  |  |  |  |
| Female | 254 | 6 | (2.4) | 248 | (97.6) | 0.58 | [0.18 - 1.72] | 0.34 |  |  |  |
| **Age** |  |  |  |  |  |  |  |  |  |  |  |
| [0 - 1 yr] | 187 | 6 | (3.2) | 181 | (96.8) | 1.00 | ref | 0.38 |  |  |  |
| [1 - 2 yr] | 233 | 7 | (3.0) | 226 | (97.0) | 0.93 | [0.31 - 2.95] |  |  |  |  |
| [2 - 3 yr] | 76 | 1 | (1.3) | 75 | (98.7) | 0.40 | [0.02 - 2.41] |  |  |  |  |
| [3 - 4 yr] | 39 | 3 | (7.7) | 36 | (92.3) | 2.51 | [0.51 - 10.0] |  |  |  |  |
| [4 - 5 yr] | 22 | 1 | (4.5) | 21 | (95.5) | 1.44 | [0.07 - 8.98] |  |  |  |  |
| **Symptoms** |  |  |  |  |  |  |  |  |  |  |  |
| Fever | 542 | 9 | (50.0) | 316 | (60.3) | 0.66 | [0.25 - 1.71] | 0.38 |  |  |  |
| Dry cough | 539 | 6 | (33.3) | 256 | (49.1) | 0.52 | [0.18 - 1.35] | 0.19 | ******* |  |  |
| Productive cough | 539 | 14 | (77.8) | 254 | (48.8) | **3.68** | **[1.30 - 13.1]** | **0.02** | **3.48** | **[1.20 - 12.6]** | **0.03** |
| Dyspnea | 542 | 15 | (83.3) | 437 | (83.4) | 1.00 | [0.32 - 4.36] | 0.99 |  |  |  |
| Chest pain | 443 | 2 | (11.8) | 24 | (5.6) | 2.23 | [0.34 - 8.55] | 0.30 |  |  |  |
| Runny nose | 540 | 14 | (77.8) | 393 | (75.3) | 1.15 | [0.40 - 4.11] | 0.81 |  |  |  |
| Sore throat | 471 | 1 | (5.9) | 35 | (7.7) | 0.75 | [0.04 - 3.84] | 0.78 |  |  |  |
| Headache | 446 | 1 | (6.2) | 22 | (5.1) | 1.24 | [0.07 -- 6.55] | 0.84 |  |  |  |
| Chills | 535 | 3 | (16.7) | 33 | (6.4) | 2.93 | [0.66 - 9.45] | 0.10 | ******* |  |  |
| Sweats | 537 | 7 | (41.2) | 111 | (21.3) | 2.58 | [0.92 - 6.87] | 0.06 | 2.08 | [0.68 - 6.06] | 0.18 |
| Anorexia | 540 | 10 | (58.8) | 233 | (44.6) | 1.78 | [0.67 - 4.96] | 0.25 |  |  |  |
| Vomiting | 542 | 2 | (11.8) | 120 | (22.9) | 0.45 | [0.07 - 1.62] | 0.29 |  |  |  |
| Diarrhea | 541 | 2 | (11.8) | 62 | (11.8) | 0.99 | [0.15 - 3.63] | 0.99 |  |  |  |
| Weight loss | 534 | 8 | (47.1) | 126 | (24.4) | **2.76** | **[1.02 - 7.36]** | **0.04** | 2.15 | [0.73 - 6.20] | 0.16 |
| Asthenia | 537 | 8 | (47.1) | 217 | (41.7) | 1.24 | [0.46 - 3.30] | 0.66 |  |  |  |
| GPD | 540 | 7 | (41.2) | 129 | (24.7) | 2.14 | [0.76 - 5.68] | 0.13 | ******* |  |  |
| Intercostal recession | 538 | 10 | (55.6) | 386 | (74.2) | 0.43 | [0.17 - 1.16] | 0.08 | ******* |  |  |
| MNW | 536 | 10 | (55.6) | 277 | (53.5) | 1.09 | [0.42 - 2.89] | 0.86 |  |  |  |
| Cyanosis | 534 | 2 | (11.1) | 74 | (14.3) | 0.75 | [0.12 - 2.70] | 0.70 |  |  |  |

GPD: general physical deterioration; MNW: movement of nose wings

^†^OR=crude odd ratio; ^‡^aOR= adjusted odd ratio; ***variables included in the initial model

**S4 Table. Comparison of Influenza A mono-infection versus *S. pneumoniae* mono-infection in Madagascar from November 2010 to July 2013**

|  |  | **Influenza A** | | ***S. Pneumoniae*** | |  |  |  |
| --- | --- | --- | --- | --- | --- | --- | --- | --- |
|  | **N** | n | (%) | n | (%) | OR^†^ | 95%CI | *p-value* |
| **Sites** |  |  |  |  |  |  |  |  |
| Cenhosoa | 26 | 14 | (53.9) | 12 | (46.1) | 1.00 | ref |  |
| Moramanga | 15 | 9 | (60.0) | 6 | (40.0) | 1.27 | [0.29 - 5.76] | 0.75 |
| **Gender** |  |  |  |  |  |  |  |  |
| Male | 20 | 8 | (40.0) | 12 | (60.0) | 1.00 | ref |  |
| Female | 21 | 15 | (71.4) | 6 | (28.6) | 3.62 | [0.86 - 16.9] | 0.06 |
| **Age** |  |  |  |  |  |  |  |  |
| [0 - 1 yr] | 14 | 8 | (57.1) | 6 | (42.9) | 1.00 | ref | 0.82 |
| [1 - 2 yr] | 16 | 9 | (56.3) | 7 | (43.7) | 0.96 | [0.22 - 4.15] |  |
| [2 - 3 yr] | 4 | 3 | (75.0) | 1 | (25.0) | 2.25 | [0.22 - 52.1] |  |
| [3 - 4 yr] | 6 | 3 | (50.0) | 3 | (50.0) | 0.75 | [0.10 - 5.37] |  |
| [4 - 5 yr] | 1 | 0 | (0 .0) | 1 | (100) | -- | -- |  |
| **Symptoms** |  |  |  |  |  |  |  |  |
| Fever | 40 | 16 | (72.7) | 9 | (50.0) | 2.67 | [0.73 - 10.4] | 0.14 |
| Dry cough | 40 | 9 | (40.9) | 6 | (33.3) | 1.38 | [0.38 – 5.26] | 0.62 |
| Productive cough | 41 | 15 | (65.2) | 14 | (77.8) | 0.54 | [0.12 - 2.11] | 0.38 |
| Dyspnea | 41 | 17 | (73.9) | 15 | (83.3) | 0.57 | [0.10 - 2.55] | 0.47 |
| Chest pain | 38 | 2 | (9.5) | 2 | (11.8) | 0.79 | [0.09 – 7.21] | 0.82 |
| Runny nose | 41 | 18 | (78.3) | 14 | (77.8) | 1.03 | [0.22 – 4.61] | 0.97 |
| Sore throat | 38 | 2 | (9.5) | 1 | (5.9) | 1.68 | [0.15 - 38.2] | 0.68 |
| Headache | 37 | 3 | (14.3) | 1 | (6.2) | 2.50 | [0.29 – 53.5] | 0.45 |
| Chills | 41 | 4 | (17.4) | 3 | (16.7) | 1.05 | [0.20 - 6.03] | 0.95 |
| Sweats | 40 | 4 | (17.4) | 7 | (41.2) | 0.30 | [0.06 - 1.24] | 0.10 |
| Anorexia | 40 | 7 | (30.4) | 10 | (58.8) | 0.31 | [0.08 - 1.11] | 0.07 |
| Vomiting | 40 | 2 | (8.7) | 2 | (11.8) | 0.71 | [0.08 - 6.50] | 0.75 |
| Diarrhea | 40 | 0 | (0.0) | 2 | (11.8) | -- | -- | -- |
| Weight loss | 40 | 4 | (17.4) | 8 | (47.1) | **0.24** | **[0.05 – 0.95]** | **0.05** |
| Asthenia | 40 | 10 | (43.5) | 8 | (47.1) | 0.87 | [0.24 - 3.08] | 0.82 |
| GPD | 40 | 5 | (21.7) | 7 | (41.2) | 0.40 | [0.09 - 1.56] | 0.19 |
| Intercostal recession | 40 | 11 | (50.0) | 10 | (55.6) | 0.80 | [0.22 - 2.80] | 0.72 |
| MNW | 40 | 9 | (40.9) | 10 | (55.6) | 0.55 | [0.15 - 1.94] | 0.36 |
| Cyanosis | 39 | 2 | (9.5) | 2 | (11.1) | 0.84 | [0.09 – 7.67] | 0.87 |

GPD: general physical deterioration; MNW: movement of nose wings

^†^OR=crude odd ratio
